# Profiling risk factors for separation of infection complications in patients with gastrointestinal and nodal diffuse large B-cell lymphoma

**DOI:** 10.1186/s12879-023-08671-5

**Published:** 2023-10-20

**Authors:** Min Xue, Zhenzhen Gao, Miaolong Yan, Yi Bao

**Affiliations:** 1 Graduate School, Bengbu Medical College, 2600 Donghai Road, Bengbu, 233000 Anhui China; 2 The Key Laboratory, The Second Affiliated Hospital of Jiaxing University, 1518 North Huancheng Road, Jiaxing, 314000 Zhejiang China; 3 The Department of Oncology, The Second Affiliated Hospital of Jiaxing University, 1518 North Huancheng Road, Jiaxing, 314000 Zhejiang China

**Keywords:** Infection complications, Risk factors, Gastrointestinal diffuse large B-cell lymphoma, Extranodal DLBCL, Nodal DLBCL

## Abstract

**Objective:**

To identify risk factors for infection complications in patients with gastrointestinal diffuse large B-cell lymphoma (GI-DLBCL) and nodal DLBCL (N-DLBCL) during treatment, respectively.

**Methods:**

Total 51 GI-DLBCL patients and 80 N-DLBCL patients were included after retrieving clinical data from a single medical center in the past ten years. Logistic regression analysis was utilized to analyze patients’ data, including baseline demographics, treatments and laboratory values, to determine independent risk factors of infection in these patients.

**Results:**

Total 28 of 51 patients (54.9%) in the GI-DLBCL group and 52 of 80 patients (65%) in the N-DLBCL group were observed infection events during treatment. A multivariate logistic regression model revealed that Ann-arbor stage IV (P = 0.034; odds ratio [OR]: 10.635; 95% confidence interval [CI]: 1.152-142.712), extra-nodal lesions ≥ 2 (P = 0.041; OR: 23.116; 95%CI: 1.144-466.949) and high serum lactate dehydrogenase (LDH) at the time of diagnosis (LDH > 252U/L; P = 0.033; OR: 6.058; 95%CI: 1.159–31.659) were independent risk factors for the development of infection in patients with GI-DLBCL after systemic treatment. In the N-DLBCL group, high serum C-reactive protein (CRP) (P = 0.027; OR: 1.104; 95%CI: 1.011–1.204) and a low platelet count (P = 0.041; OR: 0.991; 95%CI: 0.982-1.000) at routine blood tests just before infection occurred were identified as significant risk factors related to infection events during treatment.

**Conclusions:**

Discordant independent risk factors induced infection may be present during the treatment in patients with GI-DLBCL and N-DLBCL. Close monitoring these risk factors is likely an effective strategy to prevent microbial infections in these patients.

**Supplementary Information:**

The online version contains supplementary material available at 10.1186/s12879-023-08671-5.

## Introduction

Diffuse large B-cell lymphoma (DLBCL) is the most common subtype of aggressive Non-Hodgkin’s lymphomas arising from nodal as well as extranodal sites, and termed as nodal DLBCL (N-DLBCL) and extranodal DLBCL(EN-DLBCL). Gastrointestinal (GI) tract is considered be the most common extranodal involvement of DLBCLs [1, 2].

Systemic treatment is well established in the patients with DLBCL. R-CHOP regiments, including rituximab (R) plus cyclophosphamide (C), doxorubicin (H), vincristine (O), and prednisone (P) are used as a standard first-line therapy, which significantly improves overall survival (OS) of DLBCL [3, 4]. The value of consolidative radio-therapy after chemotherapy has not been widely recommended, as a recent large-scale of retrospective study suggested that DLBCL patients have excellent clinical outcomes by using chemotherapy alone without consolidative radio-therapy [5, 6]. In the treatment of GI-DLBCL, patients who received surgery followed by post-operation chemotherapy had a better prognosis compared with chemotherapy alone [7, 8]. Although approximately 60% of DLBCL patients benefit from systemic treatments, severe adverse events, such as late-onset neutropenia, post-chemotherapy pneumonia and urinary tract infection, etc., may occur, leading to reduced dosages or delayed chemo-cycles, and eventually compromise treatment efficacy [4, 5, 9, 10]. Close monitoring the possible risk factors related infection in patients with DLBCL may avoid microbial infections. However, limited studies are reported in literatures which aimed to separate the risk factors of infection between EN-DLBCL and N-DLBCL patients. Indeed, EN-DLBCL and N-DLBCL have their diverse genetic background, clinicobiological characteristics and response to therapy [8]. Therefore, the aim of this retrospective study was to identify the possible risk factors for infection in patients with GI-DLBCL and N-DLBCL during the treatment of diseases, respectively.

## Materials and methods

### Patient information and data collection

Initially, a total of 139 cases of DLBCL patients (including 52 cases of GI-DLBCL and 87 cases of N-DLBCL) were retrospectively recruited in this study who were treated in the Second Affiliated Hospital of Jiaxing University between January 2013 and March 2022. Tissue samples obtained from biopsies or surgical resections in patients with GI-DLBCL. N-DLBCL was diagnosed when the disease was confined to lymph nodes and spleen involvement. In all cases, the confirmation of diagnosis was based on the pathological examination according to the World Health Organization classification of hematopoietic and lymphoid tumors [11, 12]. All patients were staging with enhanced computed tomography (CT) scan, ultrasonography of lymph nodes, positron emission tomography/CT (PET/CT) and bone marrow biopsy. The study was approved by the Institutional Review Board of the above hospital ( No. JXEY-2022ZFYJ174), and conducted in accordance with the Declaration of Helsinki.

All the patients met the following criteria: (1) Age ≥ 18 years; (2) DLBCL patients were pathologically diagnosed based on the WHO classification [11, 12]. (3) R-CHOP regimens or only CHOP regimens without rituximab were given as the first line therapy. All patients received standard dosages every 21 days. In patients great than 80 years, reduced dosages of regimens (R-mini-CHOP, regimen with reduced to 1/2 ~ 1/3 of the standard measurement) was used.

Eight patients were excluded eventually due to meeting one of the following reasons:1) patients who diagnosed or initially treated in other hospitals; 2) Patients with a large number of missing clinical data required by this study. 3) Patients with history of thoracic radiotherapy. Finally, total 131 DLBCL patients were adopted in this study including 51 patients with GI-DLBCL and 80 patients with N-DLBCL.

**Fig. 1:**
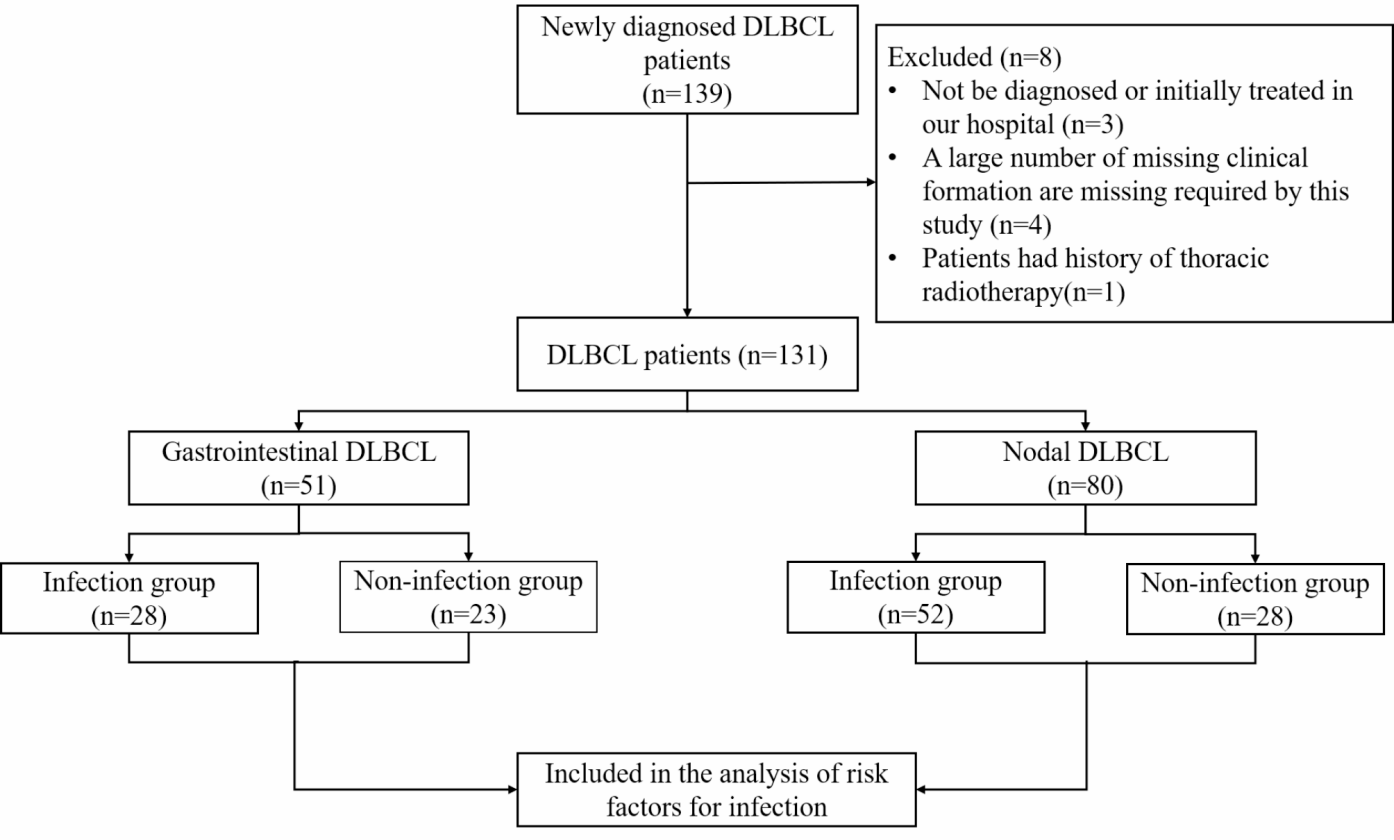
Flow chart for screening eligible patients

All DLBCL patients were further divided into infection and non-infection subgroups. Our study only counted the occurrence of first infection events. Infection in this study was diagnosed as one of the following conditions according to previously published literatures: (1) microbiologically pathogens confirmed by all kinds of cultures, such as blood or urine. (2) Pulmonary inflammatory lesions including interstitial pneumonia or signs of inflammation at an anatomic site be identified by computed tomography (CT) scan or radiography. (3) body temperature was higher than 38.0 °C during disease course, with neutrophils < 1.0 × 10^9^/L [3, 13, 14]. The granulocyte colony-stimulating factor (G-CSF) was given in patients identified neutropenia (granulocytes were below 1.0 × 10^9^/L) followed chemotherapy. In patients with severe neutropenia (granulocytes were below 0.5 × 10^9^/L), antibiotics such as cephalosporins, were given as prophylaxis against infection. The whole procedure of this study was present in Fig. 1.

To determine the risk factors for infection, the clinical characteristics (such as gender, age, Ann-arbor stage), treatments and selected hematological and biochemical parameters at the time of diagnosis (blood indicators examined before treatment) and at pre-infected laboratory examination (blood indicators examined before the start of the most recent chemotherapy cycle followed by infection complications) of 131 patients were analyzed. In patients with surgically resected GI-DLBCL, preoperative selected hematological and biochemical parameters were collected and staging was valued after operation in this study. The international prognostic index (IPI) scores were calculated 5 risk factors, including age > 60 years, stage III/IV disease, elevated lactate dehydrogenase [LDH] level, Eastern Cooperative Oncology Group [ECOG] performance status ≥ 2, two or more extranodal sites of disease [15].

### Statistical analysis

Statistical Package for the Social Sciences (SPSS), version 25.0 (IBM Inc., Chicago, IL, USA) was used for data analysis. The variables were divided into dichotomous data and continuous variables based on the types of data. Dichotomous data were analyzed using the Fisher exact test or the chi square test and continuous variables with the Wilcoxon rank sum test, as appropriate. Univariate analysis was used to determine those risk factors associated with development of GI-DLBCL and N-DLBCL infection. The factors with P < 0.2 were selected and included in the logistic regression analysis. Potential confounding factors and multicollinearity were evaluated, and the factors strongly associated with other significant factors were excluded from the multivariate analysis. Odds Ratios (ORs) and 95% Confidence Intervals (CIs) were calculated to evaluate the strength of any association. All statistical tests were two-tailed and *P* < 0.05 was considered statistically significant. The ROC curve was used to obtain the threshold values of some possible hematological and biochemical parameters for predicting infection in patients.

## Results

### Clinical characteristics of 131 DLBCL patients

The characteristics of the 131 patients with DLBCL (51 cases of GI-DLBCL patients and 80 N-DLBCL patients) are presented in Table 1. In 51 patients with GI-DLBCL, 31 (60.8%) patients were male and the median age was 67 years (range, 22–83 years). There were 37 (72.4%) GI-DLBCL patients categorized into Ann-arbor stage III-IV, and 32 (62.7%) patients had high level of IPI scores (IPI score ≥ 3). About half of the GI-DLBCL patients underwent surgical resection and postoperative chemotherapy.

**Table 1:** Clinical characteristics of the 131 DLBCL patients

	Total	GI-DLBCL	N- DLBCL
	(n = 131)	(n = 51)	(n = 80)
Sex(male)	61(46.6%)	31(60.8%)	39(48.8%)
Age(years)	66[22,85]	67[22,83]	64[23,85]
Smoking status	29(22.1%)	14(27.5%)	15(18.8%)
Hypertension	47(35.9%)	17(33.3%)	30(37.5%)
Type 2 diabetes mellitus	16(12.2%)	7(13.7%)	9(11.3%)
Ann-arbor stage			
I	11(8.4%)	9(17.6%)	2(2.5%)
II	23(17.5%)	5(10.0%)	18(22.5%)
III	55(42.0%)	16(31.4%)	39(48.8%)
IV	42(32.1%)	21(41.0%)	21(26.2%)
Bone marrow	17(13.0%)	6(11.8%)	11(13.8%)
IPI score			
< 3	58(44.3%)	19(37.3%)	37(46.2%)
≥ 3	73(55.7%)	32(62.7%)	43(53.8%)
Surgery	28(21.4%)	28(54.9%)	0
Chemo-drugs	130(99.2%)	50(98.0%)	80(100%)
Targeted drug-rituximab	65(49.6%)	23(45.1%)	42(52.5%)
Infection	80(61.1%)	28(54.9%)	52(65.0%)

Data are median and number (%). IPI, international prognostic index

Of the 80 N-DLBCL patients, 39 (48.8%) patients were male, the median age was 64 years (range, 23–85 years), 60 (75.0%) patients were categorized into Ann-arbor stage III-IV and 43 (53.8%) patients had high level of IPI scores (IPI score ≥ 3).

Infections were observed in 80 (61.1%) patients with DLBCL, including 28 patients with GI-DLBCL and 52 patients with N-DLBCL. The sites of infection are shown in Table 2. The most common site of infection was respiratory tract (63.8%), followed by blood stream infection (12.5%).

**Table 2:** All infectious episodes– Sites of infection

Sites	Number (Percent)		
	DLBCL (n = 80)	GI-DLBCL (n = 28)	N-DLBCL (n = 52)
Respiratory tract	51(63.8%)	13(46.4%)	38(73.1%)
Blood stream infection	10 (12.5%)	4(14.3%)	6(11.5%)
Gastrointestinal	1(1.2%)	1(3.6%)	0
Urogenital	7(8.7%)	4(14.3%)	3(5.8%)
Skin and Soft tissue	2(2.5%)	0	2(3.8%)
Other	9(11.3%)	6(21.4%)	3(5.8%)

Data are number (%)

### The incidence of treatment interruption/discontinuation in 131 patients with DLBCL

In 131 patients with DLBCL, total 43 of 80 (53.8%) infected patients and 4 of 51 (7.8%) non-infected patients were observed treatment interruption/discontinuation. The incidence of treatment interruption/discontinuation were significantly different between the infected group and the non-infected group (P < 0.001) (Supplementary Table 3).

### The univariate analysis of 131 DLBCL patients

In the univariate analysis, Ann-arbor stage (P < 0.001), Extra-nodal lesions (P < 0.001), ECOG score (P < 0.001), IPI scores (P = 0.027), some laboratory blood examination parameters at diagnosis including C-reactive protein (CRP), LDH and hemoglobin (Hb) (P = 0.005, P = 0.007 and 0.022), and some pre-infected laboratory indicators from blood biochemical examination before infection including the levels of CRP (P = 0.020), hemoglobin (P = 0.010), white blood cell (WBC) (P = 0.009), neutrophil/lymphocyte ratio (NLR) (P = 0.002) and LDH (P = 0.038) were statistically significant factors associating with infection in follow up. (Table 3)

**Table 3:** Baseline characteristics of infection group and non-infection group in 131 patients with DLBCL

	DLBCL				
	Total (n = 131)	Infection group (n = 80)	Non-infection group (n = 51)		P
Sex(male)	61(46.6%)	37(46.3%)	24(47.1%)		0.928
Age(years)					0.741
< 70	87(66.4%)	54(67.5%)	33(64.7%)		
≥ 70	44(33.6%)	26(32.5%)	18(35.3%)		
Ann-arbor stage					< 0.001
I	11(8.4%)	2(2.5%)	9(30.5%)		
II	23(17.5%)	7(8.7%)	16(17.4%)		
III	55(42.0%)	34(42.5%)	21(30.4%)		
IV	42(32.1%)	37(46.3%)	5(21.7%)		
Bone marrow	17(13.0%)	16(20.0%)	1(2.0%)		0.102
Extra-nodal lesions					< 0.001
< 2	101(77.1%)	53(66.3%)	48(94.1%)		
≥ 2	30(22.9%)	27(33.8%)	3(5.9%)		
ECOG score					< 0.001
0	23(17.6%)	6(7.5%)	17(33.3%)		
1	43(32.8%)	26(32.5%)	17(33.3%)		
2	32(24.4%)	22(27.5%)	10(19.6%)		
3 and 4	33(25.2%)	26(32.5%)	7(13.7%)		
IPI score					0.002
< 3	58(44.3%)	27(33.7%)	31(60.8%)		
≥ 3	73(55.7%)	53(66.3%)	20(39.2%)		
Targeted drug-rituximab	65(49.6%)	42(52.5%)	23(45.1%)		0.409
Smoking status	29(22.1%)	21(26.3%)	8(15.7%)		0.156
Hypertension	47(35.9%)	28(35.0%)	19(37.3%)		0.793
Type 2 diabetes mellitus	16(12.2%)	11(13.8%)	5(9.8%)		0.501
CRP^1^(mg/L)	15.9(3.8,44.9)	22.4(4.6,51.3)	7.5(2.5,25.2)		0.005
Hemoglobin^1^(g/L)	119.8(107,130)	117.5(107,127)	124(108,137.5)		0.022
WBC^1^ (×10^9^/L)	6.4(4.9,7.8)	6.4(4.6,7.5)	6.3(5.2,7.9)		0.711
NLR^1^	4.2(2.5,6.0)	4.5(2.4,6.0)	4.0(2.9,5.9)		0.770
Monocyte^1^(×10^9^/L)	0.5(0.3,0.6)	0.5(0.4,0.7)	0.5(0.3,0.5)		0.067
Platelet^1^(×10^9^/L)	215(157,262)	212(155,262)	222(169,260)		0.421
LDH^1^(≥ 252U/L)	73(55.7%)	52(65.0%)	21(41.2%)		0.007
CRP^2^ (mg/L)	3.9(1.7,15.3)	7.0(1.9,25.1)	3.0(1.5,8.6)		0.020
Hemoglobin^2^(g/L)	115(103,125)	112(99,123)	118.6(109.5,132)		0.010
WBC^2^(×10^9^/L)	5.1(3.4,6.6)	5.6(3.9,7.1)	4.1(3.0,5.9)		0.009
NLR^2^	3.6(2.7,7.5)	4.8(3.1,8.0)	3.0(2.3,5.5)		0.002
Monocyte^2^(×10^9^/L)	0.4(0.2,0.6)	0.4(0.1,0.6)	0.4(0.2,0.5)		0.597
Platelet^2^(×10^9^/L)	184(138,247)	173(130,222)	206(169.5,254)		0.082
LDH^2^(≥ 252U/L)	45(34.4%)	33(41.3%)	12(23.5%)		0.038

Data are median, number (%) or median and quartile, M (P25, P75). ECOG, eastern cooperative oncology group; IPI, international prognostic index; CRP, C-reactive protein; WBC, white blood cell; NLR, neutrophil/lymphocyte ratio; LDH, lactic dehydrogenase; ^1^ tests at the time of diagnosis; ^2^ tests before infection

### Independent risk factors of infectious events in 131 DLBCL patients

Factors which significant predictors of infection in the multivariable analysis included Ann-arbor stage III (P = 0.009; OR: 11.708; 95% CI: 1.868–73.376) and IV (P = 0.002; OR: 36.498; 95% CI: 3.793-351.198). Patients with DLBCL who had high LDH level at the time of diagnosis (LDH ≥ 252 U/L) had a higher risk of infection, as compared with patients with LDH < 252 U/L (P = 0.015; OR: 3.143; 95% CI: 1.248–7.916). (Table 4)

**Table 4:** Univariate analysis and multivariate analyses of risk factors for infections in 131 patients with DLBCL

	Univariate analysis		Multivariate analyses		
	P		P	OR	95%CI
Ann-arbor stage	< 0.001				
II	0.453				
III	0.017		0.009	11.708	1.868–73.376
IV	< 0.001		0.002	36.498	3.793-351.198
Extra-nodal lesions (≥ 2)	0.001				
ECOG score	0.002				
1	0.010				
2	0.003				
3 and 4	0.001				
IPI score (≥ 3)	0.003				
Smoking status	0.160				
CRP^1^(mg/L)	0.052				
Hemoglobin^1^(g/L)	0.036				
Monocyte^1^(×10^9^/L)	0.051				
LDH^1^(≥ 252U/L)	0.008		0.015	3.143	1.248–7.916
Hemoglobin^2^(g/L)	0.036				
WBC^2^(×10^9^/L)	0.048				
NLR^2^	0.537				
Platelet^2^(×10^9^/L)	0.176				
LDH^2^(≥ 252U/L)	0.039				

OR, odds ratio; CI, confidence interval; ECOG, eastern cooperative oncology group; IPI, international prognostic index; CRP, C-reactive protein; LDH, lactic dehydrogenase; WBC, white blood cell; NLR, neutrophil/lymphocyte ratio

### The univariate analysis of patients with GI-DLBCL and patients with N-DLBCL

The univariate analysis of risk factors for infection in patients with GI-DLBCL and N-DLBCL are summarized in Table 5. Among the 51 patients with GI-DLBCL, 28 (54.9%) patients experienced infection during treatment. Variables associated with the development of infection during treatment in this study were Ann-arbor stage (P = 0.014), Extra-nodal lesions (P = 0.004), IPI scores (P = 0.022), the levels of LDH at diagnosis (P = 0.013) and pre-infected WBC (P = 0.025) and pre-infected NLR (P = 0.034). However, surgical treatment was not a risk factor as no statistically significant differences were observed between infected and non-infected groups (P > 0.05).

**Table 5:** Univariate analysis of risk factors for infection in 51 patients with GI-DLBCL and 80 patients with N-DLBCL

	Gastrointestinal DLBCL (n = 51)				N-DLBCL (n = 80)		
	Infection group (n = 28)	Non-infection group (n = 23)	P		Infection group (n = 52)	Non-infection group (n = 28)	P
Sex(male)	16(57.1%)	15(65.2%)	0.557		27(51.9%)	12(42.9%)	0.870
Age(years)							0.830
< 70	23(82.1%)	17(73.9%)	0.712		31(59.6%)	16(57.1%)	
≥ 70years	5(17.9%)	6(26.1%)			21(40.4%)	12(42.9%)	
Ann-arbor stage			0.014				<0.001
I	2(7.1%)	7(30.5%)			0	2(7.1%)	
II	1(3.6)	4(17.4%)			6(11.5%)	12(42.9%)	
III	9(32.1%)	7(30.4%)			25(48.1%)	14(50%)	
IV	16(57.2%)	5(21.7%)			21(40.4%)	0	
Bone marrow	5(17.9%)	1(4.3%)	0.292		11(21.2%)	0	0.388
Extra-nodal lesions			0.004				0.053
< 2	14(48.3%)	20(90.9%)			40(76.9%)	27(96.4%)	
≥ 2	15(51.7%)	2(9.1%)			12(23.1%)	1(3.6%)	
ECOG score			0.146				0.003
0	1(3.6%)	5(21.7%)			5(9.6%)	12(42.9%)	
1	10(35.7%)	8(34.8%)			16(30.8%)	9(32.1%)	
2	7(25%)	6(26.1%)			15(28.8%)	4(14.3%)	
3 and 4	10(35.7%)	4(17.4%)			16(30.8%)	3(10.7%)	
IPI			0.022				0.005
< 3	7(25.0%)	13(56.5%)			19(36.5%)	18(64.3%)	
≥ 3	21(75.0%)	10(43.5%)			33(63.5%)	10(35.7%)	
Surgery	16(57.1%)	12(52.2%)	0.723				
Chemo-drugs	27(96.4%)	23(100%)	0.360		50(96.2%)	28(100%)	0.293
Rituximab	16(57.1%)	7(30.4%)	0.056		26(50.0%)	16(57.1%)	0.542
CRP^1^(mg/L)	32.0(9.4,62.8)	17.9(5.9,29.0)	0.176		21.2(4.2,36.7)	4.2(1.6,10.0)	0.003
Hemoglobin^1^(g/L)	116.5(109,123)	116.5(104,133)	0.820		119(103,129)	127(122.8,138)	0.006
WBC^1^ (×10^9^/L)	7.0(5.3,7.6)	6.5(5.4,7.4)	0.940		5.9(4.4,7.4)	6.0(4.8,8.1)	0.774
NLR^1^	4.5(4.1,6.0)	4.5(3.1,6.2)	0.595		3.2(2.0,6.0)	3.7(2.9,5.4)	0.353
Monocyte^1^(×10^9^/L)	0.5(0.3,0.6)	0.4(0.3,0.5)	0.477		0.6(0.4,0.8)	0.5(0.3,0.5)	0.066
Platelet^1^(×10^9^/L)	251(228,300)	231(202,264)	0.232		176(126,223)	207(142,257)	0.155
LDH^1^(≥ 252U/L)	17(60.7%)	6(26.1%)	0.013		35(67.3%)	15(53.6%)	0.226
CRP^2^ (mg/L)	5.4(1.8,15.0)	4.4(1.0,14.9)	0.583		7.9(2.1,29.7)	3.0(1.7,4.5)	0.011
Hemoglobin^2^(g/L)	111.9(101,123)	113(100,128.5)	0.449		114.1(99,122)	121.5(115,131)	0.004
WBC^2^(×10^9^/L)	5.6(4.6,8.0)	3.7(2.8,5.8)	0.025		5.6(3.8,6.8)	4.7(3.6,6.0)	0.178
NLR^2^	6.8(3.6,8.7)	3.2(2.7,6.9)	0.034		3.8(2.9,7.8)	2.9(2.1,5.1)	0.021
Monocyte^2^(×10^9^/L)	0.3(0.2,0.6)	0.3(0.2,0.4)	0.432		0.4(0.1,0.6)	0.4(0.3,0.6)	0.888
Platelet^2^(×10^9^/L)	216.7(160,300)	194(172,261)	0.513		159(112,200)	207.5(165,252)	0.015
LDH^2^(≥ 252U/L)	5(17.9%)	5(17.9%)	0.715		28(53.8%)	9(32.1%)	0.063

Data are median, number (%) or median and quartile, M (P25, P75). ECOG, eastern cooperative oncology group; IPI, international prognostic index; CRP, C-reactive protein; WBC, white blood cell; NLR, neutrophil/lymphocyte ratio; LDH, lactic dehydrogenase

52 of the 80 (65%) patients with N-DLBCL developed infection during treatment. Ann-arbor staging (P < 0.01), ECOG score (P = 0.003), IPI scores (P = 0.005) and some pre-infected laboratory indicators including NLR (P = 0.021) and platelets (P = 0.015) were statistical different between infected group and non-infected group. Moreover, Hb and CRP whether at diagnosis or before infection were significantly different between the infected group and the non-infected group in N-DLBCL patients (P < 0.05).

### The multivariate logistic regression analysis of risk factors for developing infection in GI-DLBCL patients

The multivariate logistic regression method was used to further study the risk factors for infection in GI-DLBCL patients. The results are detailed in Table 6. Ann-arbor stage IV (P =0.034; OR: 10.635; 95% CI: 1.152-142.712), Extra-nodal lesions (≥ 2) (P = 0.041; OR: 23.116; 95% CI: 1.144-466.949) and high LDH level at the time of diagnosis (LDH ≥ 252U/L; P = 0.033; OR: 6.058; 95% CI: 1.159–31.659) were identified as independent risk factors for infection in the GI-DLBCL group.

**Table 6:** Univariate and multivariate logistic regression analyses of risk factors for infections in GI-DLBCL patients

	Univariate analysis		Multivariate analyses		
	P		P	OR	95%CI
Ann-arbor stage	0.032				
II	0.923				
III	0.112				
IV	0.011		0.034	10.635	1.152-142.712
Extra-nodal lesions (≥ 2)	0.003		0.041	23.116	1.144-466.949
ECOG score	0.139				
1	0.125				
2	0.151				
3 and 4	0.022				
IPI score (≥ 3)	0.025				
Targeted drug-rituximab	0.060				
CRP^1^(mg/L)	0.107				
LDH^1^(≥ 252U/L)	0.016		0.033	6.058	1.159–31.659
WBC^2^(×10^9^/L)	0.104				
NLR^2^	0.832				

ECOG, eastern cooperative oncology group; IPI, international prognostic index; CRP, C-reactive protein; LDH, lactic dehydrogenase; WBC, white blood cell; NLR, neutrophil/lymphocyte ratio

### The multivariate logistic regression analysis of risk factors for developing infection in N-DLBCL patients

Multivariate logistic regression analysis of N-DLBCL patients was carried out and detailed in Table 7. ), Elevated pre-infected CRP (P = 0.027; OR: 1.104; 95%CI: 1.011–1.204) and low pre-infected platelet (P = 0.041; OR: 0.991; 95%CI: 0.982-1.000) were observed as independent risk factors for infection in N-DLBCL patients.

**Table 7:** Univariate and multivariate logistic regression analyses of risk factors for infections in N-DLBCL patients

	Univariate analysis		Multivariate analyses		
	P		P	OR	95%CI
Ann-arbor stage	0.214				
II	0.999				
III	0.999				
IV	0.999				
ECOG score	0.005				
1	0.041				
2	0.006				
3 and 4	0.001				
IPI score (≥ 3)	0.030				
CRP^1^(mg/L)	0.153				
Hemoglobin ^1^(g/L)	0.026				
Platelet^1^(×10^9^/L)	0.344				
CRP^2^ (mg/L)	0.013		0.027	1.104	1.011–1.204
Hemoglobin ^2^(g/L)	0.032				
WBC^2^(×10^9^/L)	0.208				
NLR^2^	0.159				
Platelet^2^(×10^9^/L)	0.029		0.041	0.991	0.982-1.000
LDH^2^(≥ 252U/L)	0.066				

ECOG, eastern cooperative oncology group; IPI, international prognostic index; CRP, C-reactive protein; WBC, white blood cell; NLR, neutrophil/lymphocyte ratio; LDH, lactic dehydrogenase

The threshold of levels of pre-infection CRP and pre-infection platelet count are 6.11 mg/L and 168 × 10^9^/L respectively (Supplementary Fig. 1).

## Discussion

Infections are common adverse events both in patients with EN-DLBCL and N-DLBCL while undergoing systemic treatment. In accordance with previous retrospective studies, the incidence of pneumonia is about 5.6-29.3% in DLBCL patients receiving chemotherapy [3, 16]. Infections often reduced the quality of patient’s life and result in poor OS eventually [3, 13]. In addition to pulmonary infections, urinary tract, gastrointestinal tract are also common sites, with even severe infections, such as sepsis. However, to our knowledge, studies are lacking in separating the independent risk factors between the patients with GI-DLBCL and N-DLBCL. For patients with GI-DLBCL, surgical resection is considered as an initial choice followed by post-operative chemotherapy, whereas patients with N-DLBCL are mainly treated with chemotherapy only. In GI-DLBCL, whether surgical procedure serves as an independent risk factor GI-DLBCL is still required to be further illuminated. Additionally, in our study, we tried to identify risk factors for infection of GI-DLBCL and N-DLBCL patients in order to remove these risk factors by earlier intervention.

Surgical resection followed by post-operative chemotherapy is considered to be a standard treatment for the management of patients with managing GI-DLBCL [17, 18]. In our study, 28 of 51 GI-DLBCL patients received surgical resection followed by chemotherapy, but there was no statistically significant difference in the development of infection between two groups of patients treated with and without surgery. Therefore, we consider that surgery is unlikely to be an independent risk factor for developing infection during GI-DLBCL treatment. But this study is based on retrospective data with a small statistical sample size, bias cannot be excluded completely. This conclusion should be further validated in a well-designed prospective study with a large sample size.

In the present study, univariate analysis revealed that in patients with GI-DLBCL, Ann-arbor staging, Extra-nodal lesions ≥ 2, IPI scores, LDH levels at diagnosis and some laboratory indicators including WBC and NLR at pre-infected laboratory examination were associated with the development of infection during treatment. Ann-arbor stage IV, Extra-nodal lesions ≥ 2 and high serum levels of LDH at the time of diagnosis were observed as independent risk factors for infection complications in GI-DLBCL patients during treatment by further multivariate logistic regression analysis in this study. DLBCL patients with advanced Ann-arbor stage (stage III/IV) have poor total physical energy status compared with patients with early stage (stage I/II), particularly in stage IV patients with bone marrow involvement who are in a high risk of developing infection due to impaired hematopoietic activities and poor immune function. According to a previous study, advanced Ann-arbor stage is an independent risk factor for pneumonia in patients with DLBCL after chemo-drugs, however this study did not separate the EN-DLBCL and N-DLBCL patients [3]. In addition, elevation of LDH level is an individual risk factor of the IPI score (IPI score ≥ 3) that is frequently used to evaluate predict the poor prognosis of non-Hodgkin’s lymphoma [14, 19]. In our study, we identified that high serum levels of LDH at the time of diagnosis were an independent risk factor for infection in GI-DLBCL patients during treatment rather than high levels of IPI scores. This observation supported by a retrospective study which was showed that elevated LDH was associated with an increased risk for developing neutropenia in patients with lymphoma after chemotherapy [13]. Elevation of serum LDH has the abilities suppressing the immune system and altering the tumor microenvironment [20].

N-DLBCL is the most common subtype of DLBCLs. The univariate analysis of patients with N-DLBCL revealed that Ann-arbor staging, ECOG score, IPI scores, CRP levels at diagnosis, Hb levels at diagnosis, and some laboratory indicators including NLR, CRP, Hb and platelets at pre-infected laboratory examination were associated with the development of infection during treatment. Further multivariate logistic regression analysis showed that high serum levels of CRP and decreased platelets at pre-infected laboratory examination were identified independent risk factors for infection in N-DLBCL treatment in our study. These data indicate that the myelosuppressive state before chemotherapy plays a key role for the development of infection in N-DLBCL patients. DLBCL patients with advanced stage are prone to develop moderate to severe myelosuppression pre- and post-treatment. We observed advanced stage in patients with N-DLBCL in this study, as approximately 75% patients diagnosed with III/IV stage. Platelets have the ability to modulate the function various immune cells and participate interaction between pathogens and host defense [21, 22]. Severe thrombocytopenia increases the probability of bacteremia, tissue damage, etc. [23]. In a previous published study, platelets less than 150 × 109/L (P = 0.002, OR: 3.67, 95%CI: 1.60–8.44) were reported to be a risk factor of febrile neutropenia in patients with DLBCL [24]. However, we preferred to present our data using platelet as a continuous variable because platelets change frequently in patients with DLBCL during treatment. The data obtained after platelets were defined as continuous variables were closer to the clinical realities and assisted the clinicians to assess the risk of infection in DLBCL patients dynamically. In addition, CRP is often used as a laboratory parameter for inflammatory diseases. It rises rapidly at the early stages of infection, which helps to earlier diagnose patients with latent infection [23]. Our study observed that elevated serum levels of CRP at pre-infected laboratory examination served as an independent risk factor for infection complications in N-DLBCL treatment. However, NLR, popular parameters used to evaluate early infection, did not showed to be independent risk factors for infection in patients with N-DLBCL in our study. This may be due to prophylactic use of G-CSF in lots of patients and their neutrophils were maintained at normal or high levels. These data supported by previous studies showing injecting G-CSF can reduce the risk of infection in cancer patients with myelosuppression after chemotherapy [25, 26]. Interestingly, Ann-arbor stage and increased serum levels of LDH did not show to be independent risk factors in N-DLBCL by multivariate logistic regression analysis, which was different from GI-DLBCL. The morbidity ratio of GI-DLBCL is lower compared to N-DLBCL due to different clinical characteristics such as early clinical stages and normal serum LDH levels in patients with GI-DLBCL [27].

In a previous study, elderly age reported to be a risk factor for pneumonia in patients with DLBCL [3]. However, inconsistent data suggested advanced age is not a dependent risk factor for infections in DLBCL patients [14]. In our study, we also did not observe elderly age serving as a risk factor in DLBCL patients. One possible reason is because of a high incidence rate of infection in both groups; additionally, R-mini-CHOP regimen with reduced dosages were used in elderly patients in current study.

A previous study reported that DLBCL patients with intermediate or greater (reference category low) IPI scores have a higher risk of infection than patients with low IPI scores [14]. But we only observed IPI scores as a risk factor in univariate analysis of all DLBCL but not in multivariate logistic regression analysis. One possible reason may be present the different variables used in statistics in two studies. Actually, the P value was very close to statistical significance, and expanding sample size may provide solutions.

One main limitation of this study is that we haven’t pointed out whether these identified risk factors for infection are also affected the survival time of DLBCL patients. Unfortunately, due to missing survival information in partial cases retrieved from our database, as well as short follow up time in partial cases, it is hard to draw a reliable conclusion currently. Nevertheless, it is important to address this question in future by further collecting data including time prolonged follow up survival time of these patients diagnosed DLBCL recently.

## Conclusions

Our study suggests that there are discordant impendent risk factors inducing infection during GI-DLBCL and N-DLBCL treatment. In GI-DLBCL patients, surgical procedure is unlikely to be an independent risk factor for developing infection during treatment. It may be valuable for monitoring risk factors for infection in GI-DLBCL and N-DLBCL separately.

### Electronic supplementary material

Below is the link to the electronic supplementary material.

Supplementary Material 1

## Data Availability

All data generated or analyzed during this study are included in this published article.

## List of abbreviations

CIs: Confidence intervals
CRP: C-reactive protein
DLBCL: Diffuse large B-cell lymphoma
ECOG: Eastern cooperative oncology group
EN-DLBCL: Extranodal diffuse large B-cell lymphoma
G-CSF: Granulocyte colony-stimulating factor
GI-DLBCL: Gastrointestinal diffuse large B-cell lymphoma
Hb: Hemoglobin
IPI: International prognostic index
LDH: Lactic dehydrogenase
N-DLBCL: Nodal diffuse large B-cell lymphoma
NLR: Neutrophil/lymphocyte ratio
ORs: Odds ratios
OS: Overall survival
PET/CT: Positron emission tomography/ computed tomography
SPSS: Statistical package for the social sciences
WBC: White blood cell
