# Supplementary material for: Profiling risk factors for separation of infection complications in patients with gastrointestinal and nodal diffuse large B-cell lymphoma
Source: BMC Infect Dis. 2023 Oct 20;23:711. doi: 10.1186/s12879-023-08671-5 (PMC10589955; doi:10.1186/s12879-023-08671-5)
Supplement: Supplementary file 1 — Supplementary Material 1 [file 12879_2023_8671_MOESM1_ESM.docx]

**Supplementary Part 1**

**Patient Collection**

A total of 75 cases of patients with extranodal DLBCL(EN-DLBCL) were retrospectively recruited in this study who were treated in the Second Affiliated Hospital of Jiaxing University in the past 5 years. Total 22 patients were excluded eventually due to meeting one of the exclusion criteria. Finally, there are 53 DLBCL patients were adopted in the study. Extranodal or extralymphatic sites include the bone marrow, gastrointestinal tract, skin, bone, central nervous system (CNS), lung, gonads, ocular adnexa (conjunctiva, lacrimal gland, and orbital soft tissue), liver, kidneys, and uterus. (PMID:30996573)

**Clinical characteristics of 53 EN-DLBCL patients.**

In 53 patients with EN-DLBCL, 30 (56.6%) patients were male and 36(67.9%) of patients were 60 years old or older. There were 38 (71.7%) EN-DLBCL patients categorized into Ann-arbor stage III-IV, and 28(52.8%) patients had high level of IPI scores (IPI score >3). About half of the EN-DLBCL patients underwent surgical resection.

**Independent risk factors of infectious events in EN-DLBCL patients**

Factors which significant predictors of infection in the multivariable analysis included Ann-arbor stage IV (P: 0.042; OR: 33.260; 95% CI: 1.134-975.648) and high LDH level at the time of diagnosis (LDH ≥252U/L; P: 0.037; OR: 4.068; 95% CI: 1.005-20.921).

**Supplementary Table 1** Univariate analysis of risk factors for infection in 53 patients with extranodal diffuse large B-cell lymphoma (EN-DLBCL).

|  | EN-DLBCL | | |  |
| --- | --- | --- | --- | --- |
|  | Total | Infection group | Non-infection group | **P** |
|  | （n=53） | （n=34） | (n=19) |  |
| Sex(male) | 30(56.6%) | 19(55.9%) | 11(57.9%) | 0.887 |
| Age (≥60 years) | 36(67.9%) | 22(64.7%) | 14(73.7%) | 0.502 |
| Ann-arbor stage |  |  |  | 0.001* |
| I | 10(18.9%) | 5(14.7%) | 5(26.3%) |  |
| II | 5(9.4%) | 1(2.9%) | 4(21.1%) |  |
| III | 22(41.5%) | 14(41.2%) | 8(42.1%) |  |
| IV | 16(30.2%) | 14(41.2%) | 2(10.5%) |  |
| IPI score |  |  |  | 0.021* |
| ＜3 | 25(47.2%) | 12(35.3%) | 13(68.4%) |  |
| ≥3 | 28(52.8%) | 22(64.7%) | 6(31.6%) |  |
| Surgery | 28(52.8%) | 18(52.9%) | 10(52.6%) | 0.983 |
| Targeted drug-rituximab | 30(56.6%) | 21(61.8%) | 9(47.4%) | 0.311 |
| CRP^1^(mg/L) | 9.2(2.3-29.7) | 10.5(2.7-33.2) | 7.9(1.7-24.3) | 0.436 |
| Hemoglobin^1^(g/L) | 116.4±19.7 | 116.3±21.6 | 116.6±17.1 | 0.948 |
| WBC^1^ (×10^9^/L) | 6.6±2.9 | 6.9±3.1 | 6.2±2.2 | 0.553 |
| Neutrophil^1^ (×10^9^/L) | 4.8±2.7 | 5.1±3.1 | 4.3±1.7 | 0.516 |
| Lymphocyte^1^(×10^9^/L) | 1.0(0.7-1.4) | 1.0(0.6-1.3) | 1.2(0.8-1.4) | 0.156 |
| NLR^1^ | 4.2(2.7-5.8) | 4.6(2.6-7.6) | 3.4(2.7-4.3) | 0.128 |
| Platelet^1^(×10^9^/L) | 233.3±99.7 | 239.1±113.1 | 222.7±68.3 | 0.738 |
| Total bilirubin^2^(umol/L) | 12.2(8.6-16.1) | 12.3(8.7-16.1) | 10.6(8.4-16.2) | 0.701 |
| LDH^1^(≥252U/L) | 26(49.1%) | 18(52.9%) | 8(42.1%) | 0.449 |
| CRP^2^ (mg/L) | 5.3(1.3-22.8) | 7.0(2.6-24.5) | 1.9(0.7-15.6) | 0.109 |
| Hemoglobin^2^(g/L) | 111.5±16.9 | 116.3±21.6 | 116.6±17.1 | 0.948 |
| WBC^2^(×10^9^/L) | 4.7(3.8-6.4) | 5.0(3.9-7.2) | 4.6(3.4-5.6) | 0.278 |
| Neutrophils^2^(×10^9^/L) | 3.1(2.0-4.7) | 3.2(2.1-5.4) | 3.1(1.9-3.8) | 0.335 |
| Lymphocyte^2^(×10^9^/L) | 0.9(0.6-1.3) | 0.7(0.4-1.2) | 0.9(0.8-1.5) | 0.086 |
| NLR^2^ | 4.0(1.8-7.1) | 5.3(2.3-8.0) | 2.4(1.8-4.4) | 0.031* |
| Platelet^2^(×10^9^/L) | 228.4±99.2 | 218.6±112.0 | 245.9±67.5 | 0.115 |
| LDH^2^(≥252U/L) | 17(32.1%) | 16(47.1%) | 1(5.3%) | 0.002* |

Data are median, number (%) or median and quartile, M (P25, P75). *P< 0.05. CRP, C-reactive protein; WBC, white blood cell; NLR, neutrophil/lymphocyte ratio. LDH, lactic dehydrogenase. ^1^ tests at the time of diagnosis. ^2^ tests before infection.

**Supplementary Table 2** Univariate and multivariate logistic regression analyses of risk factors for infections in EN-DLBCL patients.

|  | Univariate analysis |  | | Multivariate analyses | | |  |
| --- | --- | --- | --- | --- | --- | --- | --- |
|  | P |  | P | | OR | 95%CI | |
| Ann-arbor stage | 0.016 |  |  | |  |  | |
| II | 0.096 |  |  | |  |  | |
| III | 0.641 |  |  | |  |  | |
| IV | 0.002 |  | 0.042 | | 33.260 | 1.134-975.648 | |
| IPI score（≥3） | 0.024 |  |  | |  |  | |
| Lymphocyte^1^(×10^9^/L) | 0.446 |  |  | |  |  | |
| NLR^1^ | 0.129 |  |  | |  |  | |
| CRP^2^ (mg/L) | 0.157 |  |  | |  |  | |
| Lymphocyte^2^(×10^9^/L) | 0.598 |  |  | |  |  | |
| NLR^2^ | 0.029 |  |  | |  |  | |
| Platelet^2^(×10^9^/L) | 0.342 |  |  | |  |  | |
| LDH^2^(≥252U/L) | 0.010 |  | 0.037 | | 4.068 | 1.005-20.921 | |

OR, odds ratio; CI, confidence interval; CRP, C-reactive protein; LDH, lactic dehydrogenase. NLR, neutrophil/lymphocyte ratio.

**Methods**: The ROC curve was used to obtain the threshold values of levels of pre-infection CRP and platelet count for predicting infection in patients with N-DLBCL by Statistical Package for the Social Sciences (SPSS).

**Results:** The threshold of levels of pre-infection CRP and platelet count are 6.11mg/L and 168×109/L respectively.

**Supplementary Figure 1**


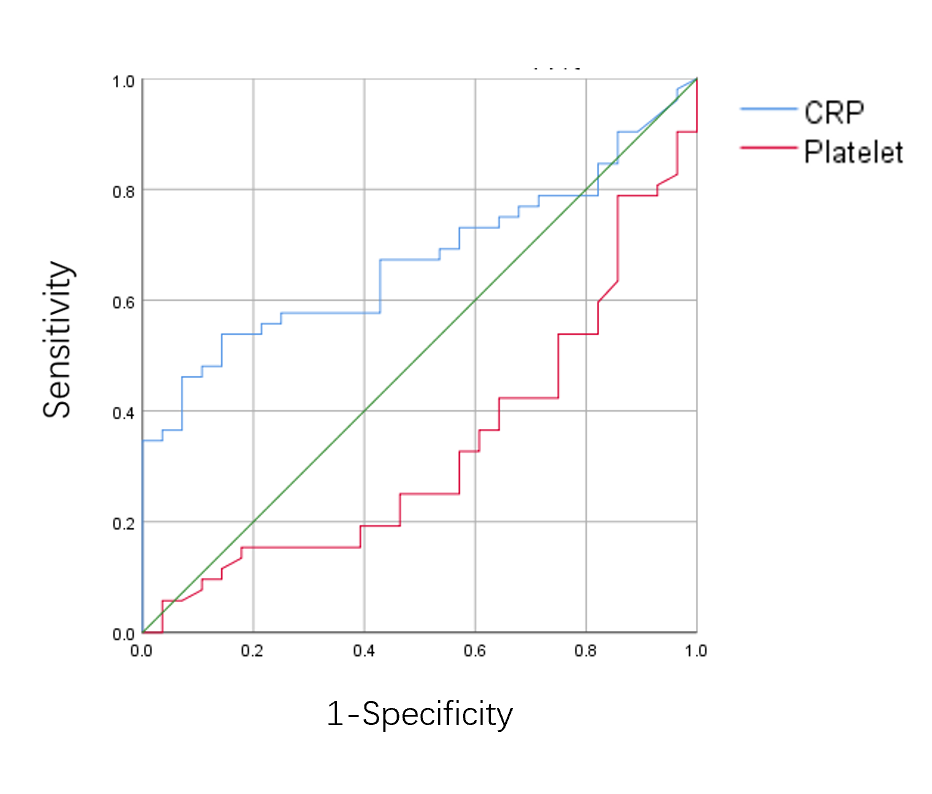


The ROC curves of pre-infection CRP and platelet count in patients with N-DLBCL

**Supplementary Table** **3** The incidence of treatment interruption/discontinuation between the infection group and the non-infection group in 131 patients with DLBCL.

|  | treatment interruption/discontinuation | | |  |
| --- | --- | --- | --- | --- |
|  | Total  (n=131) | Yes  (n=47) | No  (n=84) | P |
| Infectious events |  |  |  | < 0.001 |
| Yes | 80(61.1%) | 43(53.8%) | 37(44.0%) |  |
| No | 51(38.9%) | 4(7.8%) | 47(56.0%) |  |
